# Supplementary material for: The genome sequence of the Straw-barred Pearl moth, Pyrausta despicata Scopoli, 1763
Source: Wellcome Open Res. 2025 Mar 19;10:151. [Version 1] doi: 10.12688/wellcomeopenres.23902.1 (PMC12120437; doi:10.12688/wellcomeopenres.23902.1)
Supplement: Peer review history

# Reviewer report: Reviewer response for version 1

**DOI:** 10.21956/wellcomeopenres.26369.r121029

**Published:** 2025-05-29

**Authors:** Alan G Buddie

**Affiliations:** 1 CABI, Wallingford, England, UK

‘The genome sequence of the Straw-barred Pearl moth, *Pyrausta despicata* Scopoli, 1763’ is a very clearly written description of the genome assembly of the genome sequence of this moth which, the website ‘https://www.ukmoths.org.uk/species/pyrausta-despicata/’ confirms, is found throughout the British Isles. The ‘Background’ section notes the broader distribution of *P. despicata*, its common name, general appearance, feeding habits and reproduction mode. There is a strong justification for the publication of this genome: only one other species of the genus has had its full genome sequenced (although the mitogenome of a specimen of *P. despicata* has been described previously). Such gap-filling will help with the family-level phylogeny.

 I am certainly no expert in this field but the ’Genome sequence report’ section appears lucid, with relevant data and statistics being provided and the respective tables and figures being introduced with appropriate justification.

 The ‘Methods’ section has sufficient clarity to enable anyone, who wishes to do so, to replicate the study. The section explains that the two specimens used for HiC and HiFi sequencing, respectively, were each examined morphologically and DNA-barcoded. Whilst many of the software packages used (and the parameters mentioned) were unfamiliar to me, they seemed to have been selected with a clear and sensible rationale.

 Information on ‘Data availability’ and the ‘Author information’ are presented clearly, with appropriate (functional) web links. The reference list appears full and accurate.

 Overall, then, this manuscript appears to me to be a worthy addition to the growing list of DToL ‘gold standard’ genomes and, for me, warrants its publication in *Wellcome Open Research*. Whilst this genome note sits well on its own, its overall significance will only be fully realised when all the other species in the genus (and in the family) have been sequenced, too.

 I have couple of very minor typographical edits and two comments, but these do not detract from the very good quality of the manuscript.

Typographical edits:

 In the paragraph regarding the specimen for HiC sequencing (p6) there is a rogue ‘a’ immediately before “collected from Wytham Woods…” Please delete the ‘a’.

 There is inconsistency in the term ‘cleanup’. It is referred to, as “cleanup” or “clean up”. I would prefer to see it given as the former (or as ‘clean-up’) when referring to DNA clean-up, or similar.

Comments:

 For interested non-experts, it would be great to have terms such as ‘BUSCO’ defined – but I appreciate that this may make the manuscript considerably longer.

 For additional clarity, it would be good to have program parameters (e.g. ‘- -primary option’) given in a different font. This could also be applied usefully to software package names.

Are sufficient details of methods and materials provided to allow replication by others?

Yes

Is the rationale for creating the dataset(s) clearly described?

Yes

Are the datasets clearly presented in a useable and accessible format?

Yes

Are the protocols appropriate and is the work technically sound?

Yes

Reviewer Expertise:

Mycology, molecular biology and phylogenetics.

I confirm that I have read this submission and believe that I have an appropriate level of expertise to confirm that it is of an acceptable scientific standard.

# Reviewer report: Reviewer response for version 1

**DOI:** 10.21956/wellcomeopenres.26369.r122258

**Published:** 2025-05-10

**Authors:** Nicola J Nadeau

**Affiliations:** 1 The University of Sheffield, Sheffield, UK

There is sufficient background on the species. The methods are appropriate and the resulting genome assembly appears to be of good quality. The data and assemblies are available and accessible. Transciptome data are also available in the same project accession. The annotation using this data will increase the value to untility of this genome.

Are sufficient details of methods and materials provided to allow replication by others?

Yes

Is the rationale for creating the dataset(s) clearly described?

Yes

Are the datasets clearly presented in a useable and accessible format?

Yes

Are the protocols appropriate and is the work technically sound?

Yes

Reviewer Expertise:

Evolutionary genetics and genomics. Butterfly evolution and genetics.

I confirm that I have read this submission and believe that I have an appropriate level of expertise to confirm that it is of an acceptable scientific standard.
